# Spinal Cord Stimulation: The Clinical Application of New Technology

**DOI:** 10.1155/2012/375691

**Published:** 2011-10-05

**Authors:** Dominic Hegarty

**Affiliations:** ^1^Department of Anaesthesia and Intensive Care, Cork University Hospital, Cork, Ireland; ^2^Department of Pharmacology and Therapeutics, University College Cork, Clinical Sciences Building, Cork, Ireland

## Abstract

The use of neuromodulation for pain relief is among the fastest-growing areas of medicine, involving many diverse specialties and impacting on hundreds of thousands of patients with numerous disorders worldwide. As the evidence of efficacy improves, the interest in spinal cord stimulation (SCS) will increase because it is minimally invasive, safe, and a reversible treatment modality with limited side effect profile. While the mechanism of action evades complete understanding, the technological improvements have been considerable and current neuromodulation developments have been coupled with the rapid growth of the neuromodulation device industry resulting in the development of the next-generation neuromodulation systems. The development, the newest technicaliti and the future for the clinical application of spinal cord stimulation (SCS) are reviewed here.

## 1. Introduction

Neuromodulation is among the fastest-growing areas of medicine, involving many diverse specialties and impacting on hundreds of thousands of patients with numerous disorders worldwide [1]. Historically, electricity, either in the form of the torpedo fish or man-made electrotherapy, has been used to try and cure various ailments [2]. For example, in the middle of the 18th century “electroanalgesia” became advocated for the treatment of angina pectoris, gout, headaches, pleuritic pain, and sciatica. However, by the 20th century the enthusiasm for the medical use of electricity became associated with “quackery” [3] and was banned from clinical practice. In 1965 Melzack and Wall presented the “Gate Theory” [4], which postulated that stimulation of nonpainful stimuli can inhibit painful afference, thereby offering the opportunity to align basic research with the clinical application of electricity which has resulted in the development of neuromodulation techniques as we know them today [5–7]. While the mechanism of action evades complete understanding, the technological improvements have been considerable and current neuromodulation developments have been coupled with the rapid growth of the neuromodulation device industry resulting in the development of the next-generation neuromodulation systems. The development, the newest technicalities, and the future for the clinical application of spinal cord stimulation (SCS) are reviewed here.

## 2. Principles of Neuromodulation

Essentially there are two components of a fully implanted SCS system: the electrodes (or lead) and an implantable pulse generator (IPG). In SCS the placement of epidural electrodes is generally targeted at the dorsal column of the spinal cord; however, in patients with segmental pain (single dermatome), stimulation is focused at the corresponding dorsal root. This is where the ascending tracts pass without decussation to the gracile and cuneate nuclei of the medulla oblongata. These tracts are composed of a wide range of fiber diameters which are the central processes of the primary afferent neurons located in the spinal ganglia. As the tracts ascend, they receive accession from the dorsal roots, resulting in a somatotopic organization [6]. The recruitment of fibers is correlated directly with the diameter of the fiber and inversely with the distance between the electrode contacts and the fibers [6]. Hence the thickness of the cerebrospinal fluid layers [8], the individual anatomy, and the electrodes each influence the recruitment of the dorsal column [8–11] and dorsal root fibers [12].

In addition the large diameter fibers of the dorsal root and dorsal column have different orientation with respect to the spinal anatomy and hence in a different position in the electrical field evoked by the stimulation pulses. Furthermore, the distance between the electrode and the thickness of the dorsal cerebral spinal fluid (dCSF) layer influences the sensitivity of the fibers to the stimulation. Computer modeling predicted that a “bipolar” or a narrow guarded cathode programming sequence selectively stimulates dorsal cord fibers when the dCSF is small. In contrast, dorsal root stimulation is favored the most in “monopolar” stimulation when dCSF is wide [8–10]. In pain patients with segmental pain, stimulation can be focused on dorsal root fibers of the corresponding dermatome, whereas in patients with complex pain a multitude of dorsal column fibers related to multiple dermatomes should be stimulated. The results of these computer-based model studies led to the development of electrode arrays with similar geometric properties [13].

Once an electrode/lead is suitability positioned, the most common way to increase the intensity of the stimulation is to increase the amplitude (i.e., the current, the voltage provided); increasing the pulse rate beyond physiological limits (approximately 300 pulse per second) is not traditionally seen as providing therapeutic benefit as neural transmission may become blocked. Similarly the pulse width was traditionally set at 200 *μ*s in order to provide adequate amplitude while conserving the energy of the battery. With modern technological advancements, these concepts are now facing an interesting challenge and may influence the future of some aspects of SCS.

## 3. New Electrode Contact and Lead Design

Remarkable technological advances have been achieved in terms of electrode contact/lead design. Firstly, the new multicontact arrays available in traditional and five-column paddle leads (St. Jude Medical, Inc, USA) have resulted in the ability to provide improved programmable capability and possible treatment outcome. Mathematical modeling has highlighted the potential benefits of tight-electrode spacing in electrode contact design whereby gaps in stimulation are avoided (Boston Scientific Neuromodulation, Valencia, Calif, USA). Indeed, to obtain large paraesthesia coverage, all active contacts (anodes and cathodes at one or more arrays) should be closely spaced. 

In the beginning SCS stimulation involved only a single channel, which meant that the stimulator had only one cathodal voltage output and one anodal voltage output, each one being connected to one or more lead contacts. Only recently multichannel systems have been produced (Boston Scientific Neuromodulation, Valencia, Calif, USA). In these systems any active lead contact is driven independently with a preprogrammed current pulse. The only condition is that the sum of all cathodal and anodal currents is zero and that all pulses are synchronized. The number of settings increases exponentially from 50 combinations with four electrode contacts to tens of millions when 16 electrode contacts are available [14]. Intuitively one would be forgiven for assuming that with the newer multicontact or the multichannel systems [15] significant clinical improvements would follow, but these technical advantages have not necessarily improved treatment in all indications [16]. In fact, despite the large number of contacts available, the actual number of active contacts will generally be small (bipole, tripole, or quadruple). 

Secondly, the improved “steerability” of the leads combined with a variety of stylets to guide the positioning of the electrodes has resulted in a preference for the less invasive percutaneous insertion of the leads into the epidural space via a Touhy needle. The design of the Epiducer lead delivery system (St. Jude Medical, Inc, USA) is proposed to allow the advancement of a paddle lead without the use of a laminectomy.

Importantly, the improved flexibility of the leads has not compromised the lead fracture rate; this has fallen from 6% in earlier studies [17] to 3% [18]. Lead migration usually occurs in the first 12 months of implantation and varies between 8% [19] and 27% [20]. Migration may be related to the anchoring technique and not the actual lead design. The industry is striving to identify a solution to migration through the development of consistent and verifiable anchoring technology. Another development based on computer modeling is transverse tripolar stimulation, allowing the mediolateral steering of the electric field to correct for an inaccurate lead position [21]. Transverse tripolar steering principle led to even more complex configurations like the development of a 5-column paddle lead (Penta, St. Jude Medical).

A third technical challenge that remains is the lack of compatibility of the leads with magnetic resonance imaging (MRI) and radiofrequency diathermy which can be a significant limitation for some patients. Metallic implants (including nonferrous) are prone to heating when exposed to MRI or diathermy. In vitro comparisons showed that temperature changes near SCS electrodes were higher than those found with other metallic implants, reaching up to 4.88°C/s^−1^ [22]. While the safe use of MRI in patients with SCS leads in place has been reported [23], so too has nonreversible damage and death [20, 24]. Most manufacturers are addressing the issue, and safer leads are expected.

## 4. IPG Advancements

Originally regarded as just a battery, the IPG has now evolved to become an engineer's paradise. Long gone are the nickel-cadmium systems which are replaced by lithium-based batteries thereby prolonging the lifespan of the device. With the advent of complex stimulation settings involving the activation of an increasing number of contacts the premature exhaustion of the battery is avoided by using automatic nocturnal, time-cycled, or manual interruption of stimulation. The industry has developed a variety of new generation of compact rechargeable IPGs to meet the new requirements of SCS; thereby, energy consumption becomes less of a problem.

As previously mentioned the recruitment of dorsal horn fibers is correlated inversely with the distance between the electrode contact and the fiber [6]. Hence the thickness of the cerebrospinal fluid layers [8] the recruitment of the dorsal column [8–11] and dorsal root fibers [12]. Therefore as electrode/leads placement varies and stimulation intensity can vary depending on the position of the patient (e.g., supine or standing) to such an extent that patients cannot use the IPG without manually changing the program in order to avoid painful overstimulation.

The RestoreSensor (Medtronic Inc, USA) is the first implantable neurostimulator for spinal cord stimulation (SCS) that automatically adapts stimulation settings in response to position changes and provides objective patient activity data. The adaptive SCS is based on acceleration sensor that enables chronic motion sensing in battery-powered applications. It is robust to shock and represents the first practical, packaged, three-axis accelerometer suitable for chronic physiological measurements. The sensor's performance is also desirable for more general micropower applications like package tracking, vibration, and tilt detection.

Initial results from the Testing RestoreSensor Usability and Satisfaction (TRUST) survey [25] in 30 patients, mainly suffering from predominant leg pain due to failed back surgery syndrome or complex regional pain syndrome type I, followed up over a 10-week period are very promising. 80% of patients reported more effective pain relief. Use of the patient programmer became less difficult, and less necessary, with adaptive stimulation. Adaptive stimulation had a positive effect on sleep quality in all patients, which may have led to a perception of greater sleep quantity. In total, 58% of patients reported the ability to perform more activities with the therapy (e.g., standing, walking, sleeping, and staying in a particular position longer). Overall patient satisfaction was 97% at the end of the 10-week follow-up period [25].

## 5. Modification of the Pulse Width

In SCS, the pulse amplitude is usually the focus of stimulation control as it is intuitively understood by clinician and patient alike [26–29]. With advances in SCS technology, particularly rechargeable IPG implantable devices, pulse width (PW) programming ranges of now match that of older radiofrequency systems (with programmability up to 1000 *μ*s). Traditionally PW was only changed when other parameter adjustments fail to achieve therapeutic goals. In neurostimulation the pulse amplitude and width relate directly to the depolarization of the cell membrane and are therefore critical parameters for determining the locus of excited tissue [30]. The value of PW programming was investigated in 19 subjects who had a fully implanted SCS in place for over 3 months to treat chronic intractable low back and/or leg pain. It was shown that the baseline median PW parameter was 295 *μ*s (range 242–326 *μ*s) with a median amplitude of 2.5 mA (1.3–3.3 mA). Following independent modification of the PW, the median PW of all patients' programs increased to 400 *μ*s, approximately 48% higher (*P *= 0.01) and showed a significant increase in the paraesthesia-pain overlap (56%, *P *= 0.04). It was estimated that 10/19 patients appeared to have greater paraesthesia coverage, 7/19 patients selected the new PW programs, and 8/19 patients appeared to display a “caudal shift” of paraesthesia coverage with increased PW [31].

Mathematical modelling suggests that the mechanism behind such paraesthesia steering is due to the different selectivity of PW for larger and smaller fibers. The model considered incorporated realistic fiber size, density, and distributions in the dorsal columns, based upon human anatomic data. With a greater relative density of smaller fibers located more medial in the dorsal columns, an increase in PW will recruit smaller fibers more readily and thus result in greater midline axon recruitment. Clinically, this appeared to manifest as a caudal shift in paraesthesia. In summary variable PW programming in SCS appears to have clinical value, demonstrated by some patients improving their paraesthesia-pain overlap, as well as the ability to increase and even “steer” paraesthesia coverage [31].

## 6. High-Frequency Stimulation

Although SCS is a recommended treatment for patients with failed back surgery syndrome (FBSS) [32], if paraesthesia over the lumbar dermatomes cannot be obtained, then axial low back pain is very difficult to treat and clinical results are poor [33]. Ongoing multicentred European prospective trials [34] using dual octapolar, percutaneous leads placed sequentially near anatomic midline and connected to a rechargeable IPG capable of delivering waveforms with frequencies up to 10 kHz. (Nevro, Menlo Park, Calif) have shown that of 34 cases with full implantation the average back pain VAS decreased by 77% (8.9 cm baseline to 2.0 cm at 6 months, *P* <  .001) and leg pain VAS decreased by 82% at 6-month follow-up. (5.5 cm baseline to 0.7 cm at 6 months, *P* <  .001). In addition the average Oswestry Disability Index score decreased by 36% (from 58 to 37, *P *<  .001). This approach is novel for several reasons: (a) the use of high-frequency stimulation provides sustained analgesia in a previously difficult patient cohort without paraesthesia—thus adequate axial low back pain relief is achieved without the overwhelming leg sensation one would have expected by increasing the frequency using a traditional IPG; (b) anatomical placement of the leads is possible and intraoperative paraesthesia mapping is avoided; (c) it has decreased programming requirements; (d) continued use of the system independent of position including night-time use is possible. To date, no adverse effect of such high-frequency stimulation has been reported however, the clinical outcome in the longer term is awaited. Pre-clinical studies in goats who received 10 days of continuous stimulation at amplitudes up to the sensory/motor threshold showed no difference in the behaviour or spinal cord neural histology between the therapy and control groups. Why such stimulation has this remarkable effect still remains to be understood and may influence our approach to this co-cohort heretofore unsatisfactorily managed with conventional SCS technology.

## 7. New Clinical Applications

There are several established indications for SCS such as neuropathic back and leg pain, complex regional pain syndrome, spinal cord injury, and ischemic pain (vascular and angina pectoris). While it is beyond the remit of this paper to discuss each clinical indication, there is a growing database of clinical-based evidence to support the use of SCS. The economic evaluation in these areas is limited but the initial costs of SCS is generally both more effective and less costly then conventional management over a period of 3–5 years [33]. Unfortunately SCS is regarded as a last-resort option by many healthcare providers, and the real economic benefits may lie in the earlier introduction of the technique.

The recognition of new treatment modalities and the new application of SCS techniques will redefine our understanding of the pathophysiological concepts involved in different medical conditions. For example, painful bladder syndrome/interstitial cystitis and diffuse chronic abdominal/pelvic pain may be considered as neuropathic pain thereby offering the potential for exciting development. The evidence that cervical SCS increases cerebral blood flow (CBF) may lead to a role in cerebral ischemia. The modification of the autonomic system, particularly its sympathetic component by SCS, suggests that body functions under significant autonomic control could be subjected to modulation. It is suggested that in the future bronchospasm, gastrointestinal motility, and possibily metabolic disorders could become the focus of neuromodulation [35].

## 8. Conclusion

Modern medicine requires that any treatment modality is based on rational knowledge and well-documented theories; however, some conditions, particularly those involving chronic pain, often remain imprecise. SCS may be one of the few examples of a treatment that has significantly contributed to a change in attitudes and providing satisfactory relief to patients who in the past would have been left untreated. 

Spinal cord stimulation has significant implications for the healthcare system offering a safe reversible treatment modality with a limited side effect profile. To ensure the deliverance of a high-standard quality of care spinal cord stimulation should be provided in small well-resourced centres able to address the aftercare needs of this patient cohort. Cost-effectiveness and efficacy are fundamental if SCS is to be accepted as the therapy of choice by the public, physicians, and the healthcare decision makers. Earlier introduction of the technique may prove to be critical. Research into the mechanism of pain, the diseases, and the action of SCS requires randomized control trials (RCTs). The inability (a) to blind patients (owning to the paraesthesia), (b) to select a comparative therapy (medical, surgical, rehabilitation), and/or (c) to address the ethical implications to participate in a trial are specific issues in the design of such a RCT.

As the evidence of efficacy improves and the number of indications increases, the interest in the neuromodulation and SCS will undoubtedly increase. SCS is minimally invasive, safe, and reversible treatment modality with limited side effect profile. It is only through the combined efforts of the biomedical industry, basic science researchers, and frontline healthcare providers will the technological advancements already made in this area continue to make significant clinical impact on the patients of tomorrow.
